# Breast cancer combined with contralateral neck lymph node metastasis: a case report

**DOI:** 10.1186/s13000-022-01236-1

**Published:** 2022-07-15

**Authors:** Xiaoxiao Zhong, Fengjiao Ding, Liyuan Qian, Wei Wu, Yanguang Wen, Boni Ding

**Affiliations:** Department of Breast and Thyroid Surgery, Third Xiangya Hospital Affiliated to Central South University, Changsha, 410011 Hunan Province China

**Keywords:** Breast cancer, Contralateral neck lymph node metastasis, Treatment, Case report

## Abstract

**Background:**

Contralateral neck lymph node metastasis is rare in primary breast cancer. Its clinical staging and treatment principles lack authoritative guidelines. A case of a 30-year-old breast cancer patient with contralateral neck lymph node metastasis is presented. The clinical treatment is discussed in combination with current research.

**Case presentation:**

A 30-year-old woman presented with a right breast mass for 5 months and left neck lymph node enlargement for 5 days. Mammography showed a 33 mm*14.3 mm mass in the inner quadrant of the right breast. The ultrasound showed several hypoechoic nodules on the left side of the neck. Rapid intraoperative pathological examination diagnosed a right breast malignant tumor and poorly differentiated carcinoma of the left cervical lymph nodes. Then, right mastectomy was performed immediately. The patient was scheduled to undergo chemotherapy, molecular targeted therapy, radiotherapy and endocrine therapy after the operation. The long-term efficacy remains to be seen.

**Conclusion:**

The infrequent presentation of breast cancer with metastasis to the contralateral neck lymph node can be challenging for standard therapies.

## Background

Breast cancer is the most common malignant tumor among women. The regional lymph nodes of breast cancer refer to the axillary, supraclavicular, infraclavicular and intramammary lymph nodes on the affected side [1, 2]. The contralateral neck lymph nodes are obviously beyond the category of the regional lymph nodes and could be considered distant metastasis, belonging to stage IV. The exact drainage pathway of such metastasis is still controversial. Contralateral neck lymph node metastasis is rare for primary breast cancer, and its clinical staging and treatment principles lack authoritative guidelines. At present, scholars have no consensus on whether cervical lymph node dissection should be performed at the same time as radical mastectomy for such patients. Distant lymph node metastasis was once considered a surgical contraindication [3]. However, according to Hong Pan et al. [4], patients with distant lymph node metastases (DLNM) had similar breast cancer–specific survival (BCSS) and overall survival (OS) to patients with ipsilateral supraclavicular lymph node metastases (ISLM), and locoregional therapies were significantly associated with improved OS for patients with DLNM. Herein, we present a case of breast cancer with contralateral neck lymph node metastasis in a 30-year-old woman, exhibit its clinical, morphological, pathological and immunohistochemical characteristics and discuss its treatment combined with current research.

## Case presentation

A 30-year-old woman was admitted to the Department of Breast and Thyroid Surgery, Third Xiangya Hospital affiliated with Central South University (hereafter referred to as our hospital) on December 10th, 2020. The complaint was a right breast mass for 5 months and left neck lymph node enlargement for 5 days. The patient presented a right breast mass with the size of an egg without pain or other discomfort 5 months ago that was not treated and had not been significantly enlarged. The lymph node of the left neck was found 5 days ago, and there was no pain or discomfort. Ultrasonography revealed hypoechoic nodules in her left neck, which was considered to be a lymphadenoid tissue lesion. The patient was admitted to our hospital for further diagnosis and treatment.

Physical examination: The vital signs were normal; the neck was soft; the jugular veins were not inflated; the trachea was in the middle; and the thyroid was not enlarged. There were two palpable hard and enlarged lymph nodes approximately 1.5 cm*1.0 cm in size in the left neck, which showed poor activity. The appearance of both breasts was normal, and no “orange peel” appearance or “dimple sign” was observed on the skin. A 3.0 cm*2.5 cm hard mass was palpable in the right breast, which was irregular in shape, unclear in border, poor in motion and without tenderness. There was no palpable mass in the left breast. Bilateral axillary lymph nodes were not enlarged.

**Fig. 1:**
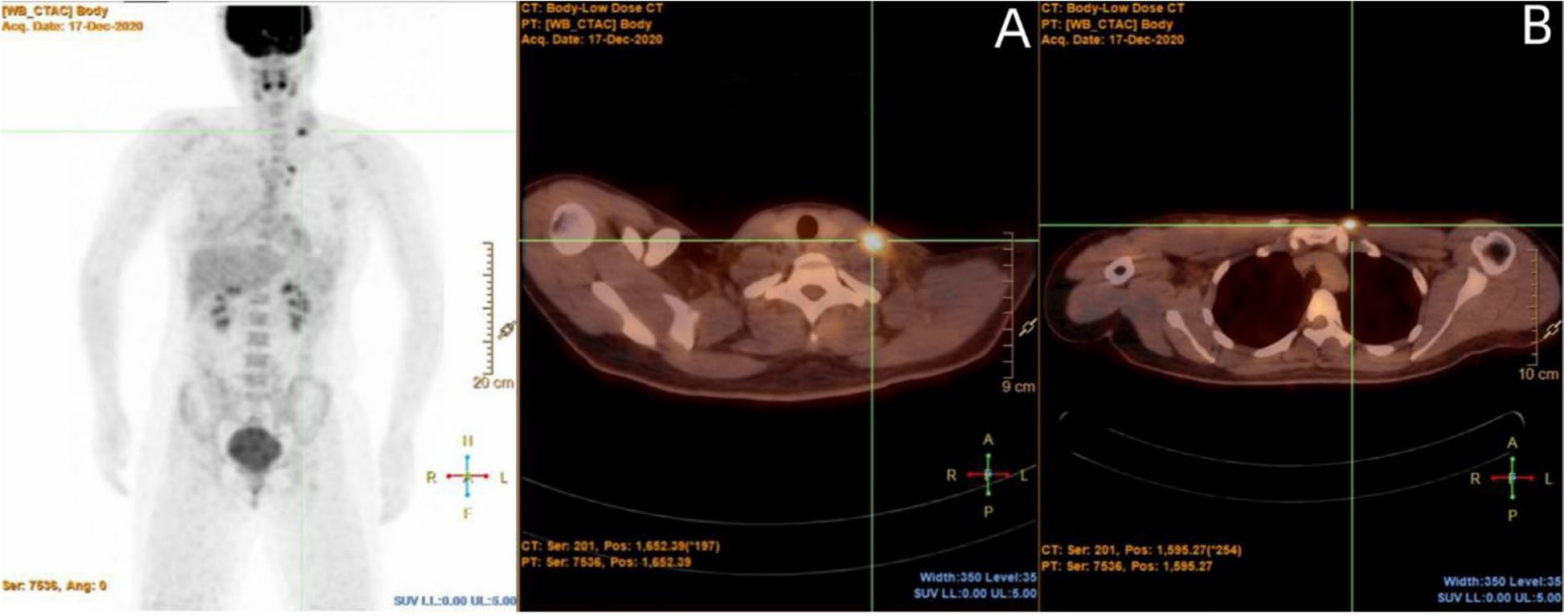
Whole-body PET-CT: There were high metabolic lymph nodes in the left neck and high metabolic nodules on the left side of the sternum stem

Laboratory examinations: Routine blood tests, liver and kidney function, tumor markers and thyroid function were normal. Ultrasound of the breast showed a hypoechoic nodule approximately 29 mm*20 mm*29 mm in size in the right breast (BR-4a). Ultrasound of the neck showed no obvious abnormal sonography of the thyroid gland. Several hypoechoic nodules on the left side of the neck were found, among which two nodules without lymphatic hila were located in cervical area III, with sizes of 20 mm*6 mm and 17 mm*9 mm. Ultrasonography of the supraclavicular lymph nodes showed no obvious swelling. The mammography showed a 33 mm*14.3 mm slightly dense mass in the inner quadrant of the right breast with fine spotty calcification (BR-4c). MRI showed a mass of 28.8 mm*23.5 mm*28.9 mm in the upper inner quadrant of the right breast (BR-5) and multiple lymph nodes in the right axilla. PET-CT (Fig. 1) showed two nodules with increased glucose metabolism in the left chest wall of the manubrium, which were considered metastases; several small lymph nodes in the left neck (region IV) accompanied by an abnormal increase in glucose metabolism were considered multiple lymph node metastases. The diagnoses were as follows: 1. right breast carcinoma; and 2. lymph node metastatic carcinoma of the left neck.

**Fig. 2:**
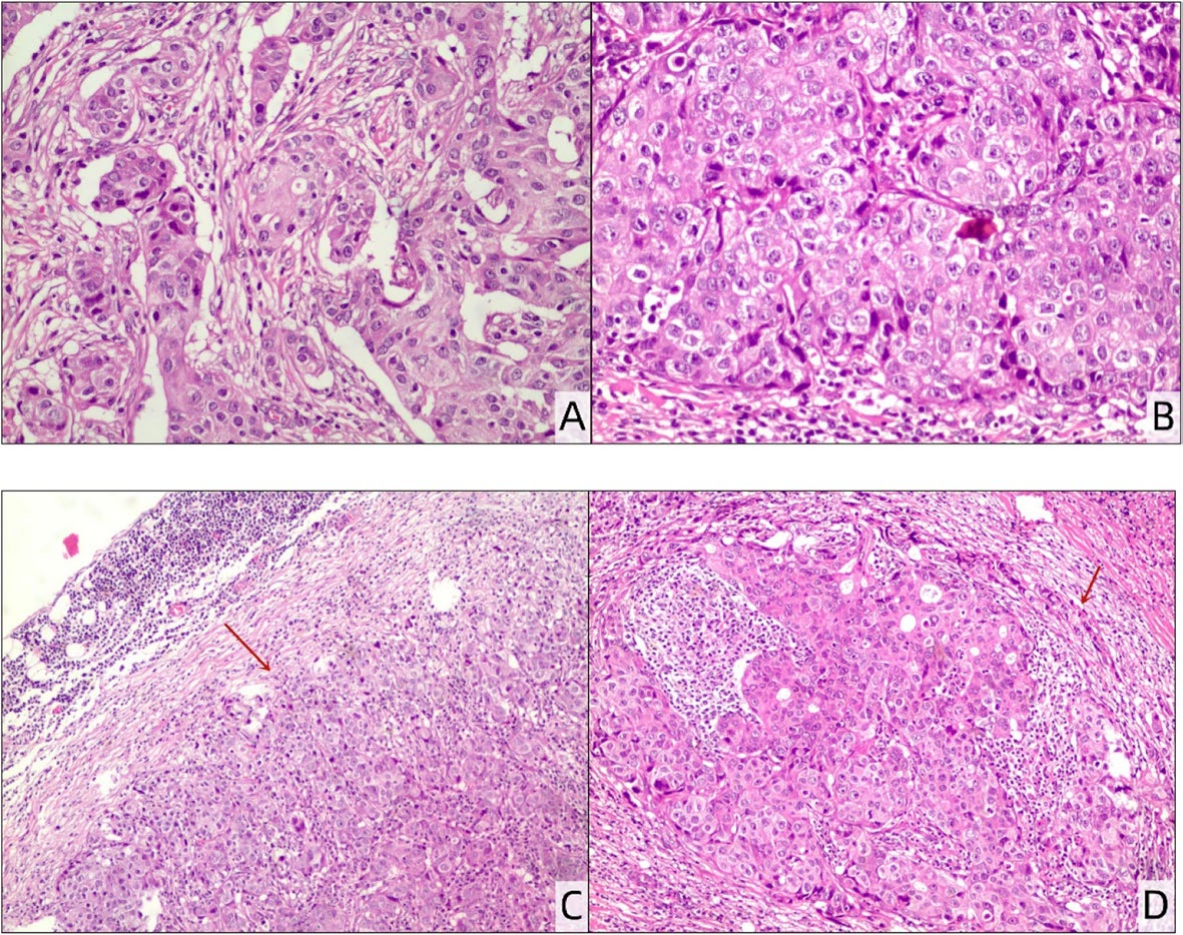
Postoperative pathological section of the right breast (**A**, **B**: H&E. 200×): invasive ductal carcinoma, grade III. Pathological section of the left neck lymph node after surgery (**C**: H&E. 40×; **D**: H&E. 100×): cancer metastasis can be seen

The preoperative preparation was completed after admission, and biopsy of the excised right breast mass as well as the left cervical lymph nodes was carried out. Rapid intraoperative pathological examination diagnosed a right breast malignant tumor and poorly differentiated carcinoma of the left cervical lymph nodes. Then, right mastectomy and sentinel lymph node biopsy of the right axilla were performed immediately. Rapid intraoperative pathological examination confirmed that there was no tumor metastasis in the right sentinel lymph node, so right axillary lymph node dissection was not performed. The postoperative pathology report (Fig. 2) showed grade III invasive ductal carcinoma of the right breast, and isolated tumor cells were found at the extracapsular vessel of one lymph node in group 1 of the sentinel lymph nodes (the number of tumor cells was less than 200). No cancer metastasis was found in the sentinel lymph nodes of groups 2, 3 and 4 (0/3). Left neck lymph node carcinoma presented metastases (2/2). No certain intravascular cancer embolus was found. Immunohistochemical examination showed ER (40%+), PR (5%+), HER-2 (3+), Ki67 (40%), AR (70%+), CK5/6 (−), CK7 (++), E-cadherin (++), p63 (−), SOX-10 (−), GATA-3 (+), Mammaglobin (+), and Napsin A (−) of the breast tumor cells, and the carcinoma cells of the left cervical lymph node were ER (70%+), PR (20%+), HER-2 (3+), Ki67 (40%), GATA-3 (+), and CK7 (++). The patient was scheduled to undergo chemotherapy, molecular targeted therapy, radiotherapy and endocrine therapy after the operation.

## Discussion

### Anatomical basis and staging of breast cancer with contralateral neck lymph node metastasis

Breast cancer with contralateral neck lymph node metastasis is very rare, and the exact drainage pathway is still controversial. The possible pathways reported in the literature include the following: 1. drainage to the posterior intercostal lymph nodes near the rib head through the cutaneous branch of intercostal vessels and then to the thoracic duct [5, 6]; and 2. skip metastasis to the contralateral axillary lymph nodes through subcutaneous lymphatic vessels in front of the sternum, through deep lymphatic vessels under thoracic fascia, or through substernal lymphatic vessels [7]. The possibility of occult breast cancer (OBC) should be excluded, while skip metastasis of breast cancer is considered. OBC should be screened through ultrasound, mammography, MRI and even PET-CT. The final diagnosis still depends on the pathological and immunohistochemical diagnosis of metastatic tumors. Although the incidence of breast cancer with contralateral cervical lymph node metastasis is relatively low, breast cancer is still considered one of the most common primary malignant tumors to metastasize to cervical lymph nodes [5, 8].

According to the American Joint Committee on Cancer (AJCC) (Eighth Edition) and Breast Cancer, Version 3.2020, NCCN Clinical Practice Guidelines in Oncology [1, 2], regional lymph nodes refer to the axillary, supraclavicular/infraclavicular and intramammary lymph nodes on the affected side. The contralateral cervical lymph nodes are obviously beyond the category of regional lymph nodes, and metastatic carcinoma of contralateral lymph nodes could be considered distant metastasis, belonging to stage IV.

### Treatment of breast cancer with contralateral neck lymph node metastasis

Breast cancer combined with contralateral neck lymph node metastasis is a clinically advanced stage and was once considered a surgical contraindication [3] because of its high rate of blood metastasis to distant organs, which is the main cause of postoperative death [9]. At present, domestic scholars have no consensus on whether cervical lymph node dissection should be performed at the same time as radical mastectomy for such patients. Bisase B et al. [9] believed that indications for cervical lymph node dissection for breast cancer included feasible local surgical treatment of breast cancer; no distant organ metastasis; intraoperative axillary lymph node metastasis but no involvement of the axillary vein; and no postoperative residual and good cervical lymph node activity without fixed fusion.

Teshome M [10] believed that the existence of primary lesions was undoubtedly the root cause of distant metastasis and increased the probability of further metastasis. Therefore, surgical treatment of a certain range should be performed as early as possible, which could reduce the tumor load and promote better therapeutic effects of radiotherapy and chemotherapy. The guidelines for diagnosis and treatment published by the National Comprehensive Cancer Network (NCCN) [1] point out that for stage IV breast cancer patients, appropriate systemic treatment should be performed according to the molecular type. The efficacy and opportunity for surgical resection of primary tumors are still being studied, and individual plans are necessary. For some patients who respond well to initial treatment, local treatment, such as breast surgery and/or radiotherapy, may be considered.

Standard electrochemotherapy (ECT) has been proven to be an effective treatment for solid tumors. A study [11] reported that lower electric field strength (LVHF ECT) and bleomycin therapies applied to recurrent breast cancer patients with neck lymph node metastasis showed successful results including local control of metastatic lymph nodes and reduction of their size, avoiding secondary surgery and reducing the adverse reactions of standard ECT. Its good efficacy, safety and tolerability make it a new treatment option for breast cancer patients with neck lymph node metastasis, especially for patients who aim to avoid secondary neck lymph node dissection after radical mastectomy.

## Conclusions

The neck lymph nodes of the patient in this case were removed during the operation. Due to the lack of intraoperative immunohistochemical diagnosis, it was not certain whether the neck metastasis was from the breast. Cervical lymph node dissection was not performed immediately. The postoperative immunohistochemical results indicated that the left neck lymph node metastasis of the patient originated from breast cancer, and mammography, ultrasound, MRI and PET-CT showed no suspicious images in the left breast. Therefore, the possibility of occult breast cancer in the left breast was temporarily ruled out. In this case, only one right sentinel lymph node was found to have isolated tumor cell micrometastases, and no metastases were observed in the right axillary lymph node, supraclavicular lymph node or contralateral axillary lymph node. Contralateral cervical lymph node metastasis was considered skip metastasis, which might be caused by metastasis to the contralateral area through subcutaneous lymphatic circulation or bypass lymphatic drainage pathways in the right breast. There is no evidence from evidence-based medicine to support whether the patient should undergo left neck lymph node dissection again. At present, the patient has been given adjuvant systemic chemotherapy, bi-target therapy, regional radiotherapy and endocrine therapy. The long-term efficacy remains to be observed.

## Data Availability

All data generated or analyzed during this case are included within the article.

## Abbreviations

DLNM: Distant lymph node metastases
ISLM: Ipsilateral supraclavicular lymph node metastases
BCSS: Breast cancer–specific survival
OS: Overall survival
OBC: Occult breast cancer
ECT: Electrochemotherapy
